# The Psychological Well-Being and Civic Engagement of Polish, Croatian and Lithuanian Academic Students during COVID-19 Outbreak

**DOI:** 10.3390/ijerph191811202

**Published:** 2022-09-06

**Authors:** Mateusz Marciniak, Sylwia Jaskulska, Slaven Gasparovic, Brigita Janiūnaitė, Jolita Horbačauskienė, Renata Glavak Tkalić

**Affiliations:** 1 School Pedagogy Research Unit, Faculty of Educational Studies, Adam Mickiewicz University in Poznan, 61-614 Poznan, Poland; 2 Department of Geography, Faculty of Science, University of Zagreb, 10000 Zagreb, Croatia; 3 Research Group in Education, Faculty of Social Sciences, Arts and Humanities, Kaunas University of Technology, 44249 Kaunas, Lithuania; 4 Ivo Pilar Institute of Social Sciences, 10000 Zagreb, Croatia

**Keywords:** well-being, civic engagement, academic students, COVID-19 outbreak

## Abstract

The aim of this research was to recognize the relationship between well-being and civic engagement under the difficult circumstances of the COVID-19 pandemic amongst students from Poland, Lithuania and Croatia. Overall, 1362 academic students (Poland, n = 596, Croatia, n = 386, and Lithuania, n = 379) participated in the study. Mean rank differences in civic engagement level (overall CE) were analysed by levels of psychological well-being (overall PWB and its subscales) using the Kruskal–Wallis test (one-way ANOVA on ranks). We conducted post hoc analysis with Bonferroni tests to measure the significance of differences in CE between the detailed levels of PWB. To avoid biases due to interaction effects between dependent variables, the analysis of mean ranks was followed by a binomial logistic regression analysis model and subgroups analysis (by gender and by country). Results obtained showed that students with higher levels of psychological well-being have higher levels of civic engagement. The differences in the CE level are most pronounced in relation to the dimension of a PWB, such as “positive relations with others”, followed by “personal growth”, “autonomy”, and “self-acceptance”. In a crisis, such as a pandemic, it is worth encouraging students to take targeted actions, as well as to create actions referring to personal development and relationships. There were no differences in the direction and shape of the associations between psychological well-being and civic engagement with respect to the country and the gender of the participants, which leads us to draw conclusions pointing to the globalised nature of student experience during the pandemic in this part of Europe.

## 1. Introduction

In psychology, well-being is defined and described in the following two ways: as a hedonistic, subjective experience of pleasure [1], or as a eudaimonistic feeling accompanying the realization of human potential [2]. The eudaimonistic approach, as argued by its promoter, Carol D. Ryff, has greater analytical potential, because it does not diagnose well-being at a given moment, as a certain effect, or as some reaction to reality. Instead, it is treated as a stable feature of a person, and this approach allows for the analysis of the course of his/her development. Research results confirm this assumption; for example, there is a strong relationship between resources of well-being in the eudaimonistic approach and a positively achieved identity, ego integration, and a sense of optimism, stable self-esteem, or empathy [3].

Among previous research on the relationship between well-being and civic engagement, there is a strong dominance of those who capture this relationship from a hedonistic perspective. Civic engagement, defined as an individual and collective, purposeful, intentional, and socially interest-oriented activity that can take many offline and online forms (from individual voluntarism to organizational involvement, to electoral participation) [4,5,6,7,8] is associated with perceived pleasure and hedonistic well-being. Supporting others, pro-environmental behaviours, and volunteering are positively related to happiness, life satisfaction, and positive affect [9]. On the other hand, the attitudes labelled as “materialism” or “consumerist orientation” are negatively related to well-being in the research [10]. Those phenomena are not opposite to civic engagement. Still, the research results in this aspect allow for a contextual interpretation of the relationship between the (non)engaged attitude and happiness or well-being.

However, there is a shortage of research that addresses the relationship between civic engagement and eudaimonistic well-being. In this approach, well-being includes characteristics of an individual, such as a balance between focusing on oneself and others, a relationship between focusing on the present and the future, and a tendency to focus on essential values [11]. Thus, it can be considered an indicator of one′s general attitude to life, so its relationship with prosocial attitudes is intriguing.

Eudaimonistic well-being is a more permanent feature of the individual, regardless of circumstances. Although both eudaimonistic well-being and hedonistic well-being undergo changes due to negative or positive experiences (illness, vacation), eudaimonistic well-being changes to a lesser extent and returns to baseline faster [12]. Therefore, it can be treated as a protective resource in challenging situations and life circumstances. For example, in the research by Carlos Freire et al., a high level of eudaimonistic well-being played a vital role in coping with stress in university students. It has been associated with using highly functional coping strategies, such as positive re-evaluation, seeking support, and planning [13].

In the present study, we focus on the relationship between civic engagement in the COVID-19 period and students’ eudaimonistic well-being in three European countries. The COVID-19 outbreak reshaped the public sphere and citizens′ participation. New areas requiring social activity have emerged, as well as new obstacles for action (e.g., restrictions). Community engagement—understood as commitment to the protection of others, attitude, leadership, or aiding in a reasonable manner—plays an essential and active role in preventing and controlling outbreaks of infectious diseases. Thus, the participation of community members (e.g., local leaders, community and faith-based organizations and groups, health facility committees, individuals, and key stakeholders) has been identified as crucial for the “bottom-up” approach used within COVID-19 responses [14]. Research results proved that offline and online engagement (e.g., shopping for people who need help, donating blood, and participating in charity events) positively influenced well-being during the lockdown. Engagement is considered a protective factor for mental health in times of crisis [15], but only under some conditions. For example, contact with others may increase one’s own fears related to the possibility of contagion. The problem is complex, as research shows, on the one hand, that engaging in helping others during a pandemic time was detrimental to mental health while, on the other hand, people who were not active (both in the area of helping others, as well as their own hobbies, free time etc.) were also at risk of mental health deterioration [16]. In the text, we focus on young adults and, in their case, as research shows, loneliness and social distance were the key factors increasing the problems in mental health. Fear of being infected was not a significant factor in this age group [17]. Considering, also, that among the factors that determine involvement is the belief in being less susceptible to infection [18], the group of young adults is one in which civic engagement during a pandemic can be seen as an essential factor for mental health protection. It should be emphasised here that people convinced that they were not exposed to the risk of COVID-19 were a group that could, nevertheless, infect others. In the text, however, we do not focus on the real results and consequences of a committed attitude, but on the relationship between well-being and civic engagement, so we are only signalling this important thread.

In our studies we concentrate on well-being using the eudaimonistic approach because we consider engagement as important not only because of the goals it pursues, but as an intrinsic, individual value [19]. This assumption makes particular sense when research concerns difficult situations [20]. Then, peoples’ individual values and assets translate into their way of experiencing this kind of situation [21,22,23].

We were interested to see how the relationship between well-being and civic engagement would be maintained under the difficult circumstances of the COVID-19 pandemic amongst students from Poland, Lithuania, and Croatia, to check how similar and/or different the experiences of students from different European countries are, thus, taking part in the discussion on the differentiating or globalizing dimension of the experience of the pandemic [24]. Additionally, we check how the well-known connection between well-being and the level of civic engagement [19] exists in the situation of pandemic crisis, and if well-being in the eudaimonistic approach is intrinsically valuable, and an if it is an individual asset to get involved in the community during the COVID-19 outbreak. This research will provide us with a better understanding of psychological well-being (in relation to civic engagement) as a protective factor during a pandemic(s) and other multidimensional crises.

## 2. Materials and Methods

### 2.1. Design

The research was conducted within cooperation from COST Action number CA17114, entitled “Transdisciplinary solutions to cross-sectoral disadvantage in youth (YOUNG-in)”, supported by COST (European Cooperation in Science and Technology). The Action CA17114 runs from 19.09.2018 to 18.03.2023 and its details are available at the following websites: https://www.cost.eu/actions/CA17114 (accessed on 1 July 2020), https://young-in.eu/ (accessed on 1 July 2020).

The online questionnaire was carried out between 14 May and 14 July 2021. To collect the survey data, the computer-assisted web interviewing (CAWI) technique (online questionnaire research) was used. The main reason for using information and communication technologies (ICT) tools was that they allow for conducting research among relatively large groups of respondents in a relatively short time. Importantly, this approach does not require direct contact between researchers and study participants, making it possible to conduct research during the COVID-19 pandemic (despite the restrictions on physical contact). However, the CAWI technique jeopardizes survey research quality to some extent and brings limitations in data interpretation and generalization.

The online questionnaire covered a series of questions, and made use of several scales, simple questions, and demographic data. Questions were taken from other authors, with the permission of the author(s) of the tool given in widespread access or direct permission, and based on the reports (mainly EUROSTUDENT [25]), and constructed by the authors of the research.

The basic version of the questionnaire was constructed in English. Afterwards, it was translated into national languages (Polish, Croatian, and Lithuanian) according to the back (reversed) translation procedure.

A pilot study preceded the main research (n = 30, academic students from diverse universities, study programs, and degrees). An analysis of the information collected within in-depth interviews was conducted during joint meetings of all members of the research team, combined with expert supervision (by researchers outside the research team). Based on the pilot study’s feedback, the final integration of the research tool was accomplished.

In the main study, potential participants received an invitation letter to participate in quantitative research. The invitation letter contained an active link to a Google form with an information sheet, full instructions, and a questionnaire.

The invitation letter was provided in a way that maximizes protection against the participation of third parties in the study. Thus, invitations were posted on the websites of faculties of universities selected for the study from Poland, Lithuania, and Croatia, or sent to students’ e-mail addresses (by faculties’ students’ offices).

Within the instructions, students received detailed information on the voluntariness of participation in the study, its purpose, duration, and fundamental rights of participants, namely anonymity, voluntariness, confidentiality, the possibility of withdrawing without giving a reason, and retention of data. We do not envisage the analysis and publication of any parts of the answers to open questions that could enable the identification of respondents. The respondents were also informed of the possibility of contacting the researchers in case of doubts or questions.

During research planning and implementation, we followed the principles of the 1964 Helsinki Declaration, and the requirements set out in this journal regarding survey studies. The research project was accepted by the Ethics Committee for Science Projects at the Faculty of Educational Studies of Adam Mickiewicz University in Poznan (No 1/16.04.2021).

### 2.2. Participants

Due to the comparative nature of the research, we decided to use the mixed sampling procedure. We selected universities and faculties for the research to provide relatively homogeneous groups of respondents (purposive sampling). The selection criteria for universities were as follows: public higher education institutions (HEIs), inclusion in the World University Rankings (Times Higher Education), a location in large academic centres in each country, and the offer of a diverse range of degrees and study programs (at International Standard Classification of Education—ISCED—level 6 or 7). The selected universities were as follows: Adam Mickiewicz University, Poznan, Poland (AMU), the University of Zagreb, Croatia (UniZG), and the Kaunas University of Technology, Lithuania (KTU). Faculties were selected (purposive sampling) where education is carried out in the disciplines of science represented at each of the three universities. Those disciplines were social, humanities, and natural sciences, according to the Organization for Economic Cooperation and Development classification [26]. The faculties at which education takes place in other science disciplines (medical and health, technical and agricultural) were excluded.

The research participants were drawn with voluntary response sampling. Academic students (n = 1872) completed the questionnaires during the research period. However, when collecting data, the responses of n = 511 students were excluded to maintain the comparability of the groups from 3 universities. The exclusion criteria were as follows: studying at a university’s satellite (branch) campus/faculty, enrolment at a part-time study program, and enrolment in a degree available only at one of the universities (e.g., speech therapy). Considering the student population of universities in 2021 [27,28,29] we determined the representative sample for each of them. Thus, with fraction size = 0.5 (50%), a maximum error of 5%, and confidence level = 95% (α = 0.95), the minimum sample size [30] for each of three universities was identified, namely 380 people (the population of AMU students in 2021-37000), 382 (UniZG-72500), and 375 (KTU-16500). After crossing the identified minimum number of academic students from each of the universities, the survey data collection was finalised (simultaneously in all countries). To avoid collecting data at different timeslots at each of the countries (which could be troublesome for data interpretation), we decided to open and close the online survey at exactly the same point in time. Thus, we did not close each country questionnaire separately after reaching its minimum sample size (minimum number of students meeting the inclusion criteria). Instead, we closed them all simultaneously after crossing the minimum sample size by the “last-loaded” country. For that reason, the research sample size exceeds the demanded minimum, which is most apparent in the case of Poland.

The final research samples of academic students (n = 1362) from the countries surveyed were as follows: Poland, n = 596 (43.8%), Croatia, n = 386 (28.3%), and Lithuania, n = 379 (27.8%). The study sample well reflected the essential demographic characteristics of university students, such as gender (female, n = 921, 67.6%; male, n = 388, 28.5%; no declaration, n = 53.3, 9%), age (M = 22.2; 18–19 year, n = 168, 12.4%; 20–21 years, n = 517, 38.0%; 22–23 years, n = 409, 30.1%; 24–25 years, n = 160, 11.8%; over 25 years, n = 105, 7.7%; no declaration n = 3, 0.2%), and living location (academic city, n = 724, 53.1%; non-academic city, n = 518, 38.0%; mixed, n = 81, 5.9%). The sample was also diverse in terms of academic characteristics, with reference to the study degree (first degree-bachelor, n = 914, 67.1%; second degree-masters, n = 357; 26.2%; integrated masters, n = 90, 6.6%), study year (1st, n = 367, 26.9%; 2nd, n = 282, 20.7%; 3rd, n = 283, 20.8%; 4th, n = 208, 15.3%; 5th, n = 200, 14.7; 6th, n = 22, 1.6%), and represented fields of science (natural, n = 634, 46.5%; social, n = 361, 26.5%; humanities, n = 338, 24.8).

### 2.3. Context

In Croatia, on 25 February 2020, the first case of COVID-19 was registered. As a response to the COVID-19 outbreak, a national health emergency was pronounced by the Croatian Government on 11 March 2020 [31]. The National Civil Defence Headquarters was established [32], and the epidemic response plan was developed by an expert group of the Ministry of Health [33]. However, the situation in Croatia was specific compared to the other countries since the well-being of Croatian citizens was seriously affected by two major earthquakes, the first one happening simultaneously with the COVID-19 outbreak. The first earthquake hit the Croatian capital Zagreb, with a magnitude of 5.5 on the Richter scale on 22 March 2020, and the second hit a smaller city, Petrinja, near the Croatian capital, with a magnitude of 6.2 on the Richter scale on 29 December 2020. There was a significant economic loss, a number of people lost their homes, and the cities were heavily damaged, resulting in a significant number of people having to relocate, all during the COVID-19 situation. Furthermore, several hospitals were damaged, causing a loss of capacity. All of this caused additional stress for Croatian citizens. Matić et al. [34] compared the level of subjective well-being among the group of people who experienced the COVID-19 outbreak and the earthquakes and a group without the earthquake experience. The result showed that people who experienced the earthquakes showed significantly decreased subjective well-being in two domains, namely standard of living and personal safety. Furthermore, people who experienced the earthquakes showed a significantly higher degree of anxiety and stress.

The first lockdown in Croatia began on March 16, 2020, with many strict restrictive measures to promote physical distancing, such as closing retail stores and restaurants, restrictions on private and public gatherings, and the transition of people to either working from home or online education [31]. Universities mostly transferred classes online for the whole of the academic years 2020/2021 and 2021/2022, with only some institutions using the hybrid form and onsite courses. The situation changed according to the recommendations from the Croatian Public Health Institute and measures implemented by the Croatian government [35]. In cases of COVID-19 infection and contact with an infected person, a social isolation measure was implemented for a period of 14 days, causing those students having onsite education to skip classes and have delays in academic activities.

Vulić-Prtorić et al. [36] examined the psychological distress among university students during eight weeks of the COVID-19 pandemic lockdown and found that the highest levels of distress occurred during the first restrictions introduced in Croatia at the beginning of the pandemic and during earthquakes that hit Croatia, but decreased due to the relaxation of all the restrictions. Pavin Ivanec [37] found that a lack of academic and social interactions was associated with more learning and self-regulation difficulties during online studying.

In Lithuania, similarly to other EU countries, after the first lockdown at the beginning of 2020, the second series of lockdowns came into effect from October 2020 on the municipality level [38], followed by a national lockdown on 7 November 2020 and an even tighter nationwide lockdown from 16 December 2020 to 31 March 2021 [39]. The tighter lockdown included very strict requirements for movement and activities, e.g., it was forbidden to leave the territory of your municipality, except when attending a funeral, for work purposes, healthcare, or when your workplace or property was located in another municipality. Non-essential travel within your municipality was forbidden. People were allowed to leave their homes to go shopping, work, attend a funeral, or seek healthcare. As for academic students, they were allowed to travel for work, such as internships or exams. Contact between more than one household was forbidden, and events involving more than one household were also banned. Public and intercity transport continued to operate, but on a reduced schedule, and wearing masks was obligatory during the period. All non-food shops had to close or move trading online, and services that involved physical contact for more than 15 min were prohibited, with exceptions applied to psychotherapy, emotional, and other health services, as well as professional legal and financial services that could not be provided remotely. As for the education system, all types of schools moved to remote classes. Universities worked in the contact form only in September 2020. Later on, with an increasing number of cases, all university activities were moved online, and the academic year 2020/2021 was finished in remote mode.

Bolatov et al. [40] investigated the influence of psychological well-being and different study formats on the academic motivation of first year medical students (N-432) during the COVID-19 pandemic in Lithuania. The results indicated that the level of psychological destruction and quality of life caused by the COVID-19 pandemic on academic motivation was minimal. It can be said that, in cases of involvement and well-being, medical students did the best, which is also confirmed by the results of research conducted in other countries, perhaps due to professional identification or greater medical knowledge [41], Zilinskas et al. [42] examined the mental health of higher education students (in different study fields) (N-1001) during COVID-19. The results indicate that the respondents highlighted anxiety and suicidality as mental health issues among higher education students in Lithuania during the COVID-19 pandemic. The study of Petkeviciute and Balciunaitiene [43] analysed the students’ experiences of learning in the remote mode during the COVID-19 pandemic in Lithuania. The study revealed that students experienced changes in the learning process, psychological problems, and a lack of knowledge and skills for creative problem-solving. At the same time, the results indicate that the respondents identified the positive aspects of remote learning while trying to solve problems that arose creatively. New hobbies, new skills, and possibilities to learn new things were mentioned as positive aspects of the lockdown period. Due to numerous limitations in professional and private functioning, the COVID-19 pandemic also changed the quality of life for many families in Poland [44,45]. This was also due to the need to switch schools and universities to remote emergency teaching and learning. Distance education in Poland before the COVID-19 pandemic was not widely practiced, and it was primarily used in relation to adults’ extramural, informal long-life learning paths than regular ones [46,47,48]. In March 2020, distance learning was introduced in Polish schools and universities by the ordinance of the Minister of National Education as a response to the need to change the way schools work during the pandemic. Students from the first three years of primary school (aged 6 to 9/10 years) had a relatively short duration of distance learning. Older students in primary school (9/10 to 15 years) and secondary school learners (aged 15 to 20), and academic students studied mainly online [49]. Most of the time, from April 2020, activities for children and teenagers under 18 were minimal e.g., only the presence of a parent, legal guardian, or an adult could justify their presence outside the home [50].

In Poland, a lot of distance learning research has been carried out since the start of the COVID-19 pandemic. Among the conclusions, the following topics play a special role: disturbed peer relations [51] and worsening health problems, especially mental health [52] or lack of digital hygiene [51]. The positive consequences of remote work in schools and universities include the improvement in teaching competency [53,54,55].

In the case of academic students in Poland during the COVID-19 outbreak, research shows worsened mental health due to the COVID-19 pandemic and the shifts in academic life that it caused [56]. The factors positively connected with mental health stability and well-being were, for example, social support or self-evaluation, and those that connected negatively included fear of COVID-19. [57]. Students also felt unprepared for the pandemic and lacked social skills and access to psychological support [58].

It is also worth mentioning that in Poland, compared to other European countries, there is low vaccine acceptance and low trust in health professionals, doctors, nurses, pharmacists, and national health authorities. This also applies to academic students [59]. There was also low resistance to following the anti-COVID recommendations [60].

### 2.4. Measures

The main research question of the research project was the following: What is the level of civic engagement of academic students during the COVID-19 outbreak and what are its determinants? The article focuses on psychological well-being as a potential determinant of civic engagement.

The main phenomena analysed within the article were measured with the following methods:

**Ryff’s Scales of Psychological Well-Being** (PWB) [61] (main predictor variable)—the scale (18-item version) authored by Carol D. Ryff. The scale measures psychological well-being and is constructed to measure its six dimensions, namely autonomy, environmental mastery, personal growth, positive relations with others, purpose in life, and self-acceptance. The modified and shortened (18-item length) version of the PWB scale was used, as it consists of 6 3-item scales (6 scales × 3 items = 18 items total). Response formats were as follows: strongly disagree (1), disagree somewhat (2), disagree slightly (3), agree slightly (4), agree somewhat (5), and strongly agree (6). The final scoring procedures covered negative scoring in the case of reversed questions. Each dimension scale scored from 3 to 18 (with overall scores from 18 to 108), while the higher the number of points, the higher the level of the PWB (overall and in terms of its dimensions). The internal consistency of the PWB scale was on satisfactory level (Cronbach’s alpha α = 0.762). The distinctions of the level of psychological well-being were derived—according to the PWB scale author’s instruction—from distributional information from the data collected as follows: high well-being referred to scores in the top 25% (4th quartile) of the distribution, low well-being scored in the bottom 25% (1st quartile) of the distribution and medium well-being was indicated by the 2nd and 3rd quartile. The scores on individual scales were combined into a composite score, which was interpreted following the above guidelines.

**Civic Engagement Scale during COVID-19** (CESC19) [5] (main predicted variable)—the scale was designed and developed by Mateusz Marciniak, based on the Civic Engagement Scale. The scale was created based on the review of related instruments, procedures, and methods used in existing civic engagement research [8,62,63,64,65,66]. The scale measures the phenomenon of civic engagement of academic students with the 10 items, divided into the 5 dimensions, namely volunteering, donation/charity, cooperation/support, activism (supports/protests), and socio-political participation. Each dimension covers two elements—one from each of two domains, as follows: (1) “COVID specific” activities—characteristic of the pandemic state, strongly connected to it or taking place in this specific period (C), and (2) non-COVID specific, usual, general activities that can be taken in normal, non-pandemic circumstances (NCS). The list of statements in dimension order was as follows:

Volunteering—I delivered meals or groceries, or otherwise supported isolated people that I know (e.g., family, neighbours) for free (statement no 1, C); I participated in volunteering activities, e.g., offered help to people in need beyond the circle of my family and friends (6, NCS).

Donation—I donated blood or personal protective equipment (e.g., face masks that I had sewn myself) to people or institutions that collected them (2, C); I donated financial resources, money or products in a social or charity fundraiser or action (7, NCS).

Cooperation—when using services or shopping, I was guided by the desire to support local entrepreneurs (e.g., Polish producers) (3, C); I cooperated with other individuals, groups, or organizations to solve the problems of a local community (8, NCS).

Activism (supports/protests)—I expressed my gratitude to healthcare and social services workers for their efforts (4, C); I signed a letter/petition or took part in a protest related to social or political issues (9, NCS).

Socio-political participation—I discussed social or political topics when meeting other people (e.g., friends, family) (5, C); I voted in the 2020 presidential/parliamentary elections (10, NCS).

The respondents indicated which of the “various activities undertaken by some people during the COVID-19 period” were undertaken by them during the last year, with the following answers: yes (1) or no (0). Each of the two subscales of civic engagement (CCE—COVID-specific engagement and NCSCE—non-COVID-specific) scored from 0 to 5, with overall scale (CE) scores from 0 to 10; the higher the number of points, the higher the level of the civic engagement domain. The internal consistency of the CE scale was on a satisfactory level (Cronbach’s alpha α = 0.704). The level of internal consistency of the CCE subscale (α = 0.643) and NCSCE subscale (α = 0.566) is questionable—it is below the threshold of α = 0.70 (the minimum value expected for the research instruments used in the social sciences). Thus, during our analyses of the relations between PWB and CE, we use only overall CE scale results and we omit CCE and NCSCE subscales results.

For better understanding of the CE scale’s structure, exploratory factor analysis (EFA) was implemented. The results of EFA showed that the sample was adequate—the Kaiser–Meyer–Olkin (KMO) Measure of Sampling Adequacy (MSA) is 0.712 (n = 1362), which can be consider as middling. Bartlett’s test of sphericity confirmed that the 10 CE items matrix is significantly different from an identity matrix, and that the variables are related to each other (chi2 = 868.175, df = 45, *p* < 0.001).The principal component analysis (PCA) showed moderate communalities of all 10 CE items (values from 0.418 to 0.677). Depending on factors retention methods it is possible to retain from 3 (with the K1—Kaiser’s method [the criteria was the eigenvalue > 1 rule]) up to 5 (cumulative percentage of variance [CPV] of 59%) constructs for rotation. The factor structure was identified with principal component analysis (the factor retention method was the K1—Kaiser’s, and the rotation metho was the Varimax with Kaiser normalization) as follows: Factor 1 (item numbers: 3, 4, 7, 1), Factor 2 (item numbers: 6, 8, 2), and Factor 3 (item numbers: 10, 5, 9). There were no major cross-loadings within factors (the primary loading of items was at least 0.200 larger than the secondary loading). The discriminant validity indicates that factors are distinct and uncorrelated on a satisfactory level (the factor correlation matrix did not reveal correlations between factors exceeding the value of 0.7, and there was no more than 50% of shared variance). The level of internal consistency of each of the three subscales created on the basis of the EFA results (corresponding with the three factors structure) was below the threshold of α = 0.70. Thus, in our analyses of relations between PWB and CE, we used only overall CE scale results.

### 2.5. Statistical Analyses

Descriptive sample analysis was performed for each variable included in the study. We describe the dependent and independent variables (PWB and CE scales and subscales) with a mean (M) and a standard deviation (SD), and with a percentage distribution (for qualitative data). We also analyse the shape of the data distribution with skewness (Sk), kurtosis (K), and the Kolmogorov–Smirnov normality test (K–S). We describe the distinctions of the level of psychological well-being (PWB) and civic engagement (CE), which were derived from distributional information from the collected data (with a range of raw data). We describe all six dimensions (subscales) and overall PWB Scale results, as well as five dimensions, two domains, and the overall CE Scale results, in the whole sample (without showing data stratified by demographic factors).

The data distribution for all civic engagement scales and psychological well-being was not normal. Thus, we used nonparametric tests to analyse the relationship between dependent (PWB) and independent (CE) variables. The mean rank differences in civic engagement level (overall CE) were analysed by levels of psychological well-being (overall PWB and its subscales) using the Kruskal–Wallis test (one-way ANOVA on the ranks). We conducted post hoc analysis with Bonferroni tests to measure the significance of the differences in CE level between the groups of students with detailed (specific) levels of PWB. To avoid biases due to interaction effects between dependent variables, the analysis of mean ranks was followed by a binomial logistic regression analysis model, and by subgroup analysis (by gender and by country). All statistical analyses were performed with 95% confidence intervals. The adopted level of significance (*p*) was α = 0.05 (1−α = 0.95). We used IBM SPSS software (statistical product and service solutions) to analyse the data.

## 3. Results

### 3.1. The Level of Students’ Psychological Well-Being

The psychological well-being of academic students was measured with Ryff’s Scale of Psychological Well-Being (PWB). As the data in Table 1 show, the research sample’s scores were diverse in relation to each of the subscales. The overall PWB among the students is high rather than low, as their average score was almost M = 80 (where 63 is the median score for the normal distribution). The same tendency applies to each of the six subscales. The skewness statistics indicate that the distribution of the data for all PWB scales is asymmetric with a mass right wing (meaning that the majority of students received high scores on the scales). The results in relation to dimensions show that the relatively lowest score was the level of student self-acceptance, and the relatively highest was personal growth.

### 3.2. The Level of Students’ Civic Engagement

As shown in Table 2, the frequency statistics of the research sample were very diverse in relation to each of the dimensions of civic engagement (CE). In general, the level of civic engagement was low rather than high. The average overall score was M = 4.2 (with standard deviation SD = 1.891 and median Me = 4), where 5.5 is the median score for the normal distribution. The data distribution for the CE Scale is not normal (Kolmogorov–Smirnov normality test, or K–S = 0.114, *p* < 0.001). The statistics for skewness (Sk = 0.136, standard error = 0.066) and for kurtosis (Kr = 0.278, standard error = 0.133) indicate that the distribution of the data for the overall CE scale is asymmetric with the mass left wing. This means that the majority of students received low scores on the CE scale. The distinction of levels of civic engagement derived from distributional information from the collected data (aimed according to quartiles of 25%, 50%, and 25%) is as follows: low CE level (scores of 0–2; n = 247, 18.1%), medium CE level (scores of 3–5, n = 774, 56.9%), and high CE level (scores of 6–10, n = 341, 25.0%).

Analysis of the dimensions of civic engagement shows that levels of volunteering and donation were relatively lowest (those form of activities were undertaken the rarest by the students). More than half of the respondents did not take part in any of the activities possible within volunteering, and the dispersion of the data within donation was similar. On the other hand, socio-political participation was the dimension with the highest level. Almost all respondents (96%) declared that they took part in at least one activity falling into the category of socio-political participation.

### 3.3. The Students’ Psychological Well-Being and Its Relationship with Civic Engagement

The analysis confirmed the statistically significant relationships between all dimensions of psychological well-being with overall civic engagement (Table 3).

Students with higher levels of psychological well-being report higher levels of civic engagement.

The dimensions of PWB are related to CE to a diverse extent. The differences in the CE level were most pronounced in relation to the PWB dimensions, such as positive relationships with others—this association was confirmed with the relatively highest value of the H statistics and the significant differences in CE level occurring between all three groups of students showing different levels of PWB. The three other PWB dimensions (personal growth, autonomy, and self-acceptance) showed average differentiation in CE (the significant differences in CE level were confirmed between groups of students showing low levels of PWB and those showing medium or high PWB). The remaining PWB dimensions (environmental mastery and purpose in life) showed the relatively lowest differentiation in CE (the only statistically significant differences in CE level occurred between students showing low and high levels of PWB).

Significant differences in CE level appear between those students who show a low level of PWB and those who show a high level of PWB (those differences apply to overall PWB and all its dimensions). There are also several significant differences between students with low and medium PWB and just one between medium and high.

### 3.4. Psychological Well-Being in Relation to Civic Engagement—Interaction Effects of Gender and Country of Origin

The associations between psychological well-being (PWB) and civic engagement (CE) were measured with mean ranks analysis. To avoid biases due to erroneous classification and intermediary effect of gender and country, we used the binomial logistic regression model for overall CE with three predictors, namely PWB, country, and gender (Table 4). For the model, the civic engagement (dependent variable) was categorised according to distributional information from the data collected. The cut-off point was 4 (the median score on CE scale and the distinction between 2 and 3 quartile). The categorization was 0 (coded for “low CE” scores from 0 to 4; n = 737; 56.3%) and 1 (coded for “high CE” scores from 5 to 10; n = 572; 43.7%), and the coefficients were estimated with maximum likelihood estimation (MLE).

The relations between the students’ civic engagement and their PWB, gender, and country of origin as a whole fit significantly better than an empty model (n = 1309; Wald test: W = 20.687, *p* < 0.001; likelihood ratio LR: ch2 = 172.408, df = 4; Nagelkerke R2 = 0.114, df = 4, *p* < 0.001,). There were no differences in the direction and shape of the associations between civic engagement and psychological well-being with respect to the country and the gender of the participants. In the analysed model, gender was the crucial factor, followed by country and psychological well-being. When psychological well-being increases, the relative likelihood odds of being in a reference category (low CE) decline by 0.361. When all other independent factors remain constant, the increasing values of the level of PWB correspond with a 43.5% increasing odds ratio of the academic student being in “high CE” category.

We followed logistic regression analysis by mean rank analyses in subgroups (subsets by gender and by country) to check if the relationships between PWB and CE remained unrevised.

Regarding male students (n = 388), the significant effects of overall PWB and CE associations remained unrevised (H = 19.261, *p* < 0.001). In the case of female students (n = 921), the association between the level of overall PWB and CE also turned out to be statistically significant (H = 7.479, *p* < 0.05).

The mean rank analysis by country confirmed the shape of all relations between PWB and CE. The significant effects remained unrevised regarding all three countries, as follows: Poland (n = 596; H = 12.825, *p* < 0.001), Lithuania (n = 379; H = 18.586, *p* < 0.001), and Croatia (n = 387; H = 7.237, *p* < 0.05).

The analysis confirmed that there are no differences in the direction and shape of the relationships between psychological well-being and civic engagement of academic students with respect to their gender and country. The relations between PWB and CE were stable and remained unrevised while analysing them in subsets of participants by gender (male and female) and by country.

## 4. Discussion

Our study demonstrates existing disparities in the distribution of civic engagement among academic students with diverse psychological well-being. The greater the value of one, the greater the value of the other. This result is consistent with the research conducted so far. Usually, these relationships concern hedonistic well-being, so the conclusions support the observation that increasing civic activity is related to increased well-being [66]. In our research, we examined the relationship between eudaimonistic well-being and civic engagement, and this relationship has also been statistically confirmed.

Academic students in our research have well-being at an average level. The detailed results regarding PWB allow us to conclude that students had the highest average in the personal growth dimension and the lowest in environmental mastery.

Our research results also indicate that the overall level of civic engagement of academic students is low. Volunteering and donation were the forms of civic engagement that students participating in our research used less often. To simplify things, we can assume that 6 out of 10 students have not participated in any volunteering activities during the 12 month COVID-19 outbreak. Almost half of the students have not participated in any form of donation or charity. Only slightly more than half of them took part in some form of cooperation. The situation for activism was the same.

The results comply with the findings of other researchers who also found low or average levels of civic engagement in academic students during a pandemic outbreak [67,68]. One of the explanations could be the life changes due to the COVID-19 pandemic. Many activities have clearly been impeded by the pandemic (e.g., on campus and in the workplace), and facing such an uncertain environment brought some decreases within the dimension of related activities [68,69]. The diverse forms of CE (not including socio-political participation) were on a similar, low level. This is also in line with the findings of the research of other authors—at the average level, the university community, local, and global civic engagement were on a similar level [68].

In contrast, in our study, the vast majority (over three out of four) students took part in both socio-political participation activities (which were elections and discussions about social and political issues). A similar effect was observed in the US 2020 elections. The college-aged voters also turned out in record numbers for the 2020 election and, what is more, the numbers were higher than in the previous elections (in non-pandemic state) [70]. The reasons for the observed increase in voting participation in the US and other countries might be somehow related to the COVID-19 outbreak (e.g., introduction of new voting options, psychological well-being of the community, distress levels, etc.). However, it might also be related to specific country circumstances (e.g., student activism due to “racial injustice” or voter suppression, and global citizenship due to climate change, etc.).

An important clue for the interpretation is also that the group that “makes the difference” in CE diversity is the group with the lowest level of PWB. The group of students with a lower level of PWB is the most different from others (students with a medium and high level of PWB) in relation to civic engagement. That means that a high, or even average level of well-being is a protective factor, while students with low well-being cope significantly worse, statistically. The results of other studies indicate that people with low well-being are not only statistically significantly different from those with medium and high scores, but also that those who report low well-being are more susceptible to a larger increase through participation, so such activities would have a greater impact on their functioning compared to people with high and medium well-being levels [71,72].

In the case of the dimensions of PWB, the most vital relationship with CE was identified as positive connections with others, followed by personal growth, autonomy, and self-acceptance. Other studies also show prosocial behaviours, high-quality relationships, and belonging to peer groups in the community context [69,73] as being connected with civic engagement. For example, according to *p*. A. Arvanitidis, in cases of academic students researched by her, a connection between having good relations based on trust and civic engagement is evident [74]. In cases of young people, researchers also report the importance of friendship and support in civic engagement [75]. The relation between self-reflective attitudes and civic engagement is also shown in the research [76,77]. In light of the research results, autonomy turns out to be a significant factor as it is positively related with youths’ levels of civic responsibility and engagement [76]. Relationships between personal growth and civic engagement are also diagnosed, for example in such a way that various types of projects and interventions in groups of young people positively contributed to both personal growth and civic engagement [78,79].

Considering our findings, the relationship between psychological well-being and civic engagement during the COVID-19 pandemic occurs independently of other factors (e.g., gender and country). Therefore, the high level of well-being may be a stronger factor than other characteristics, but the diagnosed trend may also be related to the pandemic situation that has globalised the experiences of young people in Europe. It is worth repeating the research concerning PWB and CE in the post-pandemic period.The conducted research has several limitations which might have impacted research findings. We point out some of those disadvantages for further improvements:

*Data Collection Method*

We used the CAWI method, and the links to the survey were sent with ICT. This could influence research sample findings on two levels. Firstly, the online survey excludes students with limited Internet access, which could affect sampling. Secondly, the researched phenomenon (civic engagement) also covers online activities, so limited access to the Internet might significantly shape it.

*Research Sampling*

We opened and closed online questionnaires simultaneously to avoid data collection in different timeslots for each country. This affected the size of each country’s sample—there are significantly more students from Poland than from Croatia and Lithuania. It may bias overall results in case of cross-country differences.

*Measures Design*

Both phenomena analysed within the article—PWB and CE—were measured with research tools which deliver subjective information. Academic students reported their well-being and engagement, but it was not confirmed with corresponding activities (e.g., frequency of behaviours). It reduces possible interpretations and recommendations. The CESC19 scale’s reliability level is satisfactory for overall results, but it is questionable for the subscales. It is recommended to interpret and analyse the overall results rather than the results on the subscales. The CESC19 needs further development.

*The Researched Area and the Scope of Discussion*

The article focuses on the relationship between psychological well-being and civic engagement during the COVID-19 outbreak. The relationships between phenomena were more robust (more vital) concerning COVID-specific activities than regular (general) engagement. The possible interpretations of the results of research findings are limited by the lack of previous research in the field. The longitudinal analysis of the well-being and civic engagement with repeated observations (before/during/after?) the COVID-19 outbreak would allow for a much better understanding of the nature of the analysed relations.

## 5. Conclusions

The main aim of our study was to examine the relationship between well-being and civic engagement in the time of the COVID-19 pandemic. Research findings allow us to better describe well-being as a protective factor during a pandemic and other social crises.

According to our assumptions, engagement attitude is important in one’s life not only because of the goals it pursues, but as an intrinsically individual value and, in this sense, it can relate to eudaimonistic well-being. It turned out that the relationship is the most robust (most vital) link between CE and the dimensions of a PWB, such as positive relations with others, followed by personal growth, autonomy and self-acceptance. Therefore, in a crisis, such as a pandemic, it is worth encouraging students to taking care of their relationships (in an appropriate, compliant with pandemic restrictions manner), as well as to create actions referring to personal growth to use the assets of eudaimonistic well-being.

## Figures and Tables

**Table 1:** Descriptive results of the study—assessment of psychological well-being during the COVID-19 outbreak in the experiences of Polish, Croatian, and Lithuanian students. (**a**) Raw results on the Ryff’s PWB Scale; M = mean, SD = standard deviation, Me = median, Sk = skewness [standard error = 0.066], Kr = kurtosis [standard error = 0.133], K–S = Kolmogorov–Smirnov normality test [for all K–S statistics *p* < 0.001]. (**b**) Distinctions of the level of well-being derived from distributional information from the collected data (aimed according to the quartiles of 25%, 50%, and 25%). Here, n = 1362.

PWB Scales	(a) Scale statistics						(b) Distinctions of Level of Well-Being Dimensions								
							Low			Medium			High		
	M	SD	Me	Sk	Kr	K–S	Score	n	%	Score	n	%	Score	n	%
Autonomy	13.10	2.471	13	−0.209	−0.229	0.097	1–11	345	25,3	12–14	601	44.1	15–18	416	30.5
Environmental mastery	12.25	2.687	12	−0.402	0.162	0.099	1–10	327	24.0	11–14	752	55.2	15–18	283	20.8
Personal growth	14.75	2.238	15	−0.650	0.375	0.138	1–13	384	28.2	14–16	656	48.2	17–18	322	23.6
Positive relations with others	13.28	2.876	14	−0.473	−0.105	0.101	1–11	373	27.4	12–15	655	48.1	16–18	334	24.5
Purpose in life	13.85	2.683	14	−0.679	0.299	0.129	1–12	376	27.6	13–15	580	42.6	16–18	406	29.8
Self-acceptance	12.40	3.506	13	−0.591	−0.234	0.122	1–9	289	21.2	10–14	628	46.1	15–18	445	32.7
Overall PWB	79.64	11.323	81	−0.392	0.005	0.054	41–72	351	25.8	73–87	647	47.5	88–108	364	26.7

**Table 2:** Descriptive results of the study—the level of civic engagement during the COVID-19 outbreak in the experiences of Polish, Croatian, and Lithuanian students (n = 1362). (a) Raw results on the Civic Engagement Scale during COVID-19; frequency of “Yes” and “No” responses regarding the CESC19 scale’s items (item numbers in order of dimensions; for items’ content see measurement section). (b) Distinctions of the level of civic engagement derived from distributional information from the collected data, as follows: low (0) = lack of “yes” responses regarding any of the items from a specific CE dimension, medium (1) = one “yes” response regarding items from a specific CE dimension, high (2) = both “yes” responses regarding items from a specific CE dimension.

CESC19 Dimensions	(a) Scale Statistics (Frequency)					(b) Distinctions of Levels of Civic Engagement Dimensions					
	Item Numbers	Yes		No		Low (0)		Medium (1)		High (2)	
		n	%	n	%	n	%	n	%	n	%
Volunteering	1 (C)	491	36.0	871	64.0	794	58.3	453	33.3	115	8.4
	6 (NCS)	192	14.1	1170	85.9						
Donation	2 (C)	159	11.7	1203	88.3	726	53.3	558	41.0	78	5.7
	7 (NCS)	555	40.7	807	59.3						
Cooperation	3 (C)	779	57.2	583	42.8	531	39.0	732	53.7	99	7.3
	8 (NCS)	151	11.1	1211	88.9						
Activism	4 (C)	481	35.3	881	64.7	549	40.3	561	41.2	252	18.5
	9 (NCS)	584	42.9	778	57.1						
Socio-polit. participation	5 (C)	1176	86.3	186	13.7	55	4.0	258	18.9	1049	77.0
	10 (NCS)	1180	86.6	182	13.4						

**Table 3:** Dependent results of the study—students’ psychological well-being during the COVID-19 outbreak in the experiences of Polish, Croatian, and Lithuanian students versus their civic engagement—results of the Kruskal–Wallis test (one-way ANOVA on ranks); Here, Mr = mean rank, K–W test = Kruskal–Wallis H test, CE = civic engagement, and L-M, L-H, M-H = results of post hoc analysis with Bonferroni tests—mentioned differences in CE mean ranks between the detailed levels of PWB (L-M = low level of PWB vs. medium PWB level; L-H = low vs. high, M-H medium vs. high) are statistically significant (all *p* < 0.05); (n = 1362).

The Level of PWB (by Dimensions)			The Level of CE (Overall)	
PWB Scales	Level	*N*	Mr	K–W Test
Autonomy	Low	345	611.12	H = 15.170 df = 2 *p* < 0.001 (L-M, L-H)
	Med	601	705.22	
	High	416	705.61	
Environmental mastery	Low	327	649.15	H = 5.474 df = 2 *p* < 0.05 (L-H)
	Med	752	680.10	
	High	283	722.60	
Personal growth	Low	384	595.68	H = 28.674 df = 2 *p* < 0.001 (L-M, L-H)
	Med	656	701.25	
	High	322	743.61	
Positive relations with others	Low	373	584.13	H = 41.046 df = 2 *p* < 0.001 (L-M, L-H, M-H)
	Med	655	692.11	
	High	334	769.43	
Purpose in life	Low	376	640.30	H = 6.586 df = 2 *p* < 0.05 (L-H)
	Med	580	688.28	
	High	406	709.96	
Self-acceptance	Low	289	630.32	H = 6.410 df = 2 *p* < 0.05 (L-M, L-H)
	Med	628	693.25	
	High	445	698.16	
Overall PWB	Low	351	600.58	H = 23.716 df = 2 *p* < 0.001 (L-M, L-H)
	Med	647	693.22	
	High	364	738.70	

**Table 4:** Results of binomial logistic regression for the relationship between civic engagement (CE) during the COVID-19 outbreak in the experiences of Polish, Croatian, and Lithuanian students and factorial models, namely psychological well-being (PWB), gender, and country; B = coefficient; SE = standard error; W = Wald statistic; Exp(B) = the odds ratio.

Factors	The Binomial Logistic Regression of CE and Multiple of Factors				
	B	SE	W	*p*	Exp(B)
PWB	0.361	0.082	19.251	<0.001	1.435
	Gender (Female were reference category)				
Male	−0.671	0.133	25.632	<0.001	0.511
	Country (Poland was reference category)				
Croatia	−0.060	0.136	0.191	0.662	0.942
Lithuania	−1.117	0.150	55.290	<0.001	0.327
Cons.	−0.495	0.185	7.170	<0.01	0.610

## Data Availability

The data presented in this study are available on request from the author who carries out the analyses (M.M.). The data are not publicly available due to their containing information that could compromise the anonymity of research participants.
